# Association Between the Patient Driven Payment Model and Therapy Utilization and Patient Outcomes in US Skilled Nursing Facilities

**DOI:** 10.1001/jamahealthforum.2021.4366

**Published:** 2022-01-07

**Authors:** Momotazur Rahman, Elizabeth M. White, Brian E. McGarry, Christopher Santostefano, Peter Shewmaker, Linda Resnik, David C. Grabowski

**Affiliations:** 1 Department of Health Services, Policy & Practice, Brown University School of Public Health, Providence, Rhode Island; 2 Department of Medicine, University of Rochester, Rochester, New York; 3 Department of Health Care Policy, Harvard Medical School, Boston, Massachusetts

## Abstract

**Importance:**

In October 2019, Medicare changed its skilled nursing facility (SNF) reimbursement model to the Patient Driven Payment Model (PDPM), which has modified financial incentives for SNFs that may relate to therapy use and health outcomes.

**Objective:**

To assess whether implementation of the PDPM was associated with changes in therapy utilization or health outcomes.

**Design, Setting, and Participants:**

This cross-sectional study used a regression discontinuity (RD) approach among Medicare fee-for-service postacute-care patients admitted to a Medicare-certified SNF following hip fracture between January 2018 and March 2020.

**Exposures:**

Skilled nursing facility admission after PDPM implementation.

**Main Outcomes and Measures:**

Main outcomes were individual and nonindividual (concurrent and group) therapy minutes per day, hospitalization within 40 days of SNF admission, SNF length of stay longer than 40 days, and discharge activities of daily living score.

**Results:**

The study cohort included 201 084 postacute-care patients (mean [SD] age, 83.8 [8.3] years; 143 830 women [71.5%]; 185 854 White patients [92.4%]); 147 711 were admitted pre-PDPM, and 53 373 were admitted post-PDPM. A decrease in individual therapy (RD estimate: −15.9 minutes per day; 95% CI, −16.9 to −14.6) and an increase in nonindividual therapy (RD estimate: 3.6 minutes per day; 95% CI, 3.4 to 3.8) were observed. Total therapy use in the first week following admission was about 12 minutes per day (95% CI, −13.3 to −11.3) (approximately 13%) lower for residents admitted post-PDPM vs pre-PDPM. No consistent and statistically significant discontinuity in hospital readmission (0.31 percentage point increase; 95% CI, −1.46 to 2.09), SNF length of stay (2.7 percentage point decrease in likelihood of staying longer than 40 days; 95% CI, −4.83 to −0.54), or functional score at discharge (0.04 point increase in activities of daily living score; 95% CI, −0.19 to 0.26) was observed. Nonindividual therapy minutes were reduced to nearly zero in late March 2020, likely owing to COVID-19–related restrictions on communal activities in SNFs.

**Conclusions and Relevance:**

In this cross-sectional study of SNF admission after PDPM implementation, a reduction of total therapy minutes was observed following the implementation of PDPM, even though PDPM was designed to be budget neutral. No significant changes in postacute outcomes were observed. Further study is needed to understand whether the PDPM is associated with successful discharge outcomes.

## Key Points

**Question:**

Was the Patient Driven Payment Model (PDPM), implemented in October 2019, associated with rehabilitation therapy utilization and health outcomes of patients admitted to skilled nursing facilities (SNFs)?

**Findings:**

In this cross-sectional study of 201 084 patients admitted to an SNF after hip fracture between January 2018 and March 2020, those admitted post-PDPM received about 13% fewer therapy minutes than those admitted pre-PDPM, but the likelihood of rehospitalization and functional scores at discharge remained unchanged.

**Meaning:**

Implementation of PDPM was associated with a reduction in the volume of therapy use without changes in subsequent hospitalization risk or discharge functional scores.

## Teaser

This cross-sectional study assesses whether implementation of the Patient Driven Payment Model is associated with changes in therapy utilization or health outcomes.

## Introduction

Rehabilitation services delivered in skilled nursing facilities (SNFs) are a key component of patient recovery from a hospitalization stay. Roughly 20% of hospitalized Medicare patients are discharged to an SNF for postacute care,^1^ with the vast majority of these patients receiving rehabilitative physical, occupational, and/or speech therapy services. From 1998 to October 2019, Medicare per diem payments to SNFs were largely determined by the amount of therapy delivered under the Resource Utilization Group (RUG) system, creating strong financial incentives for SNFs to deliver high amounts of therapy. Between 2002 and 2015, the share of SNF days classified as intensive therapy days (at least 500 minutes of therapy over a 7-day period) increased from 29% to 82%, while the share of days in the highest “ultrahigh” case mix group (at least 720 therapy minutes over a 7-day period) increased from 49% to 57% of total days.^2^ These efforts contributed to substantial growth in Medicare spending on SNF care, despite a lack of evidence supporting the clinical value of the additional therapy.^3,4,5^

In an effort to alter these incentives, the Centers for Medicare & Medicaid Services (CMS) implemented the Patient Driven Payment Model (PDPM) in October 2019 to replace the RUG system. Per diem payments under the PDPM are no longer linked to therapy minutes. Instead, they are determined by patients’ clinical and functional characteristics at the time of their SNF admission.^6^ Previous research has demonstrated that these altered incentives resulted in modest cuts in therapy staffing levels by SNFs during the first quarter of program implementation.^7^ However, the association between the PDPM and the amount and type of therapy delivered to Medicare SNF patients is unknown. Furthermore, the association of the PDPM with key patient outcomes, such as length of SNF stay, risk of rehospitalization, and improvement in activities of daily living (ADL) score, is not known. Therefore, the purpose of this study was to examine the association of PDPM implementation with therapy use and postacute outcomes through the first 2 quarters of 2020. We focused primarily on patients admitted to an SNF following hospitalization for hip fracture to isolate the association of PDPM and therapy use separate from any changes in the types of patients SNFs may have admitted following program implementation.

## Methods

This study was approved by the Brown University institutional review board, which waived the requirement for participant informed consent owing to human participant exemption category 4. This study followed the Strengthening the Reporting of Observational Studies in Epidemiology (STROBE) reporting guidelines for cohort studies (eAppendix in the Supplement).

### Data Sources

We used the 2017 to 2020 Minimum Data Set (MDS) and Medicare Beneficiary Summary File (MBSF). Under the RUG system, assessments were completed within 5 days of admission; at days 14, 30, 60, and 90 of SNF stay and quarterly thereafter; and at discharge. Under PDPM, CMS reduced the frequency of the required MDS assessments. However, the 5-day scheduled assessment is mandatory under both payment regimens, thereby providing a common assessment point to evaluate the PDPM. The MBSF includes enrollment and clinical information on Medicare beneficiaries and monthly Medicare Advantage enrollment indicators.

### Study Population

Our sample consisted of fee-for-service Medicare beneficiaries 65 years and older who were admitted to an SNF between January 1, 2018, and June 30, 2020, and had a 5-day scheduled MDS assessment. Patients with a previous nursing facility stay in the 1-year period prior to the admission date were excluded. Finally, our primary analyses focused on patients with hip fracture because they are commonly discharged to SNFs for postacute care^8,9,10^ with substantial therapy needs. We also examined all fee-for-service enrollees with a 5-day scheduled MDS assessment in a secondary analysis.

### Study Variables

#### Main Exposure

The main exposure was SNF admission occurring on or after October 1, 2019, when the PDPM took effect, vs admission pre-PDPM between January 1, 2017, and September 30, 2019. Specifically, we included time as a daily variable that is set to zero at October 1, 2019, and ranges between −638 and 182 owing to the cohort selection window.

#### Therapy Utilization

One of the primary outcomes of this study was therapy use, measured as the total combined number of physical therapy, occupational therapy, and speech therapy minutes provided in the preceding 7 days, as reported on the 5-day MDS assessment. We examined individual and nonindividual (concurrent and group) therapy minutes separately as well as their total. We focused on the reported minutes on the 5-day assessment, which is mandatory under both payment systems. Because 5-day assessment can be completed from days 1 to 8, we divided the reported therapy minutes by the number of days between admission date and assessment date.

#### Patient Outcomes

We examined 3 patient outcomes focusing on the first 40 days of nursing home stay. First, we identified hospitalizations from the SNF within 40 days of SNF admission based on the MDS discharge assessments. Although hospitalizations are typically identified using claims data, we relied on the MDS because claims data corresponding to the study period were not yet available, and about 94% of SNF hospitalization events in Medicare claims can be identified using MDS 3.0 discharge records.^11^ Second, we measured length of stay as a binary indicator of stays more than 40 days using discharge date. Third, we measured the change in patients’ ADL scores at admission and discharge.

#### Other Measures

Patients’ age, sex, and race and ethnicity were determined from the MBSF. This information was originally obtained from the Master Beneficiary Record of the Social Service Administration and then supplemented by additional information from CMS.^12^ The categories for race and ethnicity were Black, Hispanic, White, and other race. To capture clinical complexity at the time of SNF admission, we included the ADL score, Cognitive Function Scale score,^13^ Changes in Health, End-stage disease and Symptoms and Signs (CHESS) score,^14^ and a series of diagnoses and symptoms captured in the MDS.

### Statistical Analysis

We started by checking the balance of observations pre-PDPM and post-PDPM. We first assessed how the likelihood of having a 5-day assessment and the number of days between admission and 5-day assessment changed following PDPM implementation. We also assessed whether demographic composition and patient acuity at admission changed under PDPM.

To assess the overall changes in outcomes following the implementation of PDPM, we used a regression discontinuity (RD) approach. We first plotted measures with respect to SNF admission date to assess any discontinuity in the trend following the PDPM. We also fit RD models, estimating outcomes as a function of continuous time and its fourth-degree polynomials and an indicator of admission post-PDPM. In this specification, the coefficient of the post-PDPM indicator captures any discrete jump in trend associated with PDPM. Other variables included were age, sex, race, calendar-month dummies, day-of-the-week dummies, and SNF fixed effects. We did not include MDS-reported comorbidities and acuity measure because acuity measures might be overreported following PDPM because they are used to determine payment. The 95% CIs are based on clustered errors by SNF. There are 2 important properties of this model. First, we did not interact time with the PDPM indicator because we aimed to estimate the change in average outcomes following PDPM. Such interactions are typically included in interrupted time series models and not RD models. Second, we started our study period on January 1, 2018, which allowed us to observe individuals admitted in a given month for multiple years and incorporate month fixed effects. We chose this approach because we anticipated seasonal variation in the acuity of newly admitted SNF patients, and we wanted to isolate PDPM from the usual shift owing to the start of the fall/winter season. For similar reasons, we also included day-of-the-week fixed effects.

To assess the changes in therapy minutes, we started by comparing the distribution of therapy minutes reported in 5-day assessment pre-PDPM and post-PDPM implementation (ie, October 1, 2019, and later). For the RD in therapy minutes, we included the number of days between admission and 5-day assessment as a control variable. For the RD in discharge ADL score, we included admission ADL score as a control variable. Similarly, we plotted ADL score trajectories before and after PDPM over the first 40 days of SNF stay using all ADL reporting assessments. Analyses were performed using Stata, version 17 (StataCorp LLC).

### Supplementary Analyses

We conducted several supplementary analyses. First, we examined therapy use including interim assessments (in addition to the 5-day assessment). Second, we examined all newly admitted SNF patients with a 5-day assessment to assess whether the results that we observed in the hip fracture sample held for the entire sample. Third, we examined whether the results varied substantially across different types of SNFs, specifically, by profit status, chain affiliation, and the share of Medicare-paid residents. Fourth, we examined how sensitive our RD estimate was depending on the degree of polynomials of time in our model (we used fourth-degree polynomials in our main specification). Finally, we estimated models using observations in 2 different time windows: January 2019 to June 2020 (9 months before and after PDPM was implemented) and April 2019 to March 2020 (6 months before and after PDPM was implemented).

## Results

### Study Population

We started with more than 5 million Medicare beneficiaries newly admitted to a nursing home between January 2018 and June 2020. We identified 201 084 fee-for-service Medicare beneficiaries who were admitted to an SNF with a hip fracture and had a 5-day scheduled MDS assessment (see eTable 1 in the Supplement for sample size with different inclusion criteria). Of these, 147 711 patients were admitted pre-PDPM, and 53 373 were admitted post-PDPM. Following PDPM, the number of SNF admissions per day (eFigure 1 in the Supplement) and proportion of individuals with a 5-day assessment (eFigure 2 in the Supplement) remained roughly constant. However, we observed a discontinuous drop in days between admission date and the date of 5-day assessment (eFigure 3 in the Supplement) and the number of MDS assessments during the first 40 days of a nursing home stay (eFigure 4 in the Supplement).

**Figure 1.**
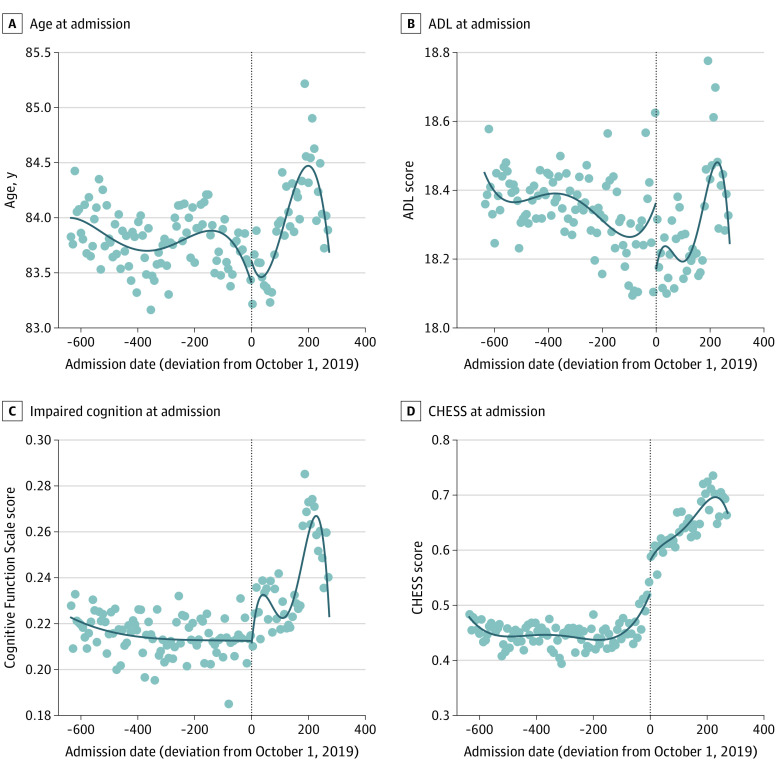
Characteristics of Patients Admitted to a Skilled Nursing Facility With Hip Fracture Diagnosis Each panel is based on a separate regression discontinuity plot. Each scatter dot represents a 7-day average. The lines are based on fourth-degree polynomials. Age, activities of daily living (ADL), and cognitive functioning regression discontinuity estimates are not statistically significant. Impaired cognition was defined as a Cognitive Function Scale score of 3 or 4. The Changes in Health, End-stage disease and Symptoms and Signs (CHESS) regression discontinuity estimate is 0.1 (95% CI, 0.07-0.13).

Table 1 shows that among patients admitted before the PDPM, the mean (SD) age was 83.8 (8.3) years, and 106 080 (71.8%) were women; 5362 (3.6%) were Black patients; 1739 (1.2%) were Hispanic patients; 136 461 (92.4%) were White patients; and 4105 (2.8%) were patients of other races (includes American Indian or Alaska Native, Asian, and Native Hawaiian or Other Pacific Islander). The demographic composition remained roughly the same after the PDPM was implemented, with mean (SD) age of 83.9 (8.3) years and 37 750 (70.7%) women; 1955 (3.7%) were Black patients; 572 (1.1%) were Hispanic patients; 49 393 (92.6%) were White patients; and 1430 (2.7%) were patients of other races. Figure 1A shows that mean age at admission declined over the study period, but there was no trend break with PDPM implementation.

**Table 1.** Characteristics of Patients With Hip Fractures Admitted to a Skilled Nursing Facility Before and After the Patient Driven Payment Model (PDPM)

Characteristic	Patients, No. (%)	
	Before PDPM (n = 147 711)	After PDPM (n = 53 373)
**Demographic characteristics**		
Age, mean (SD), y	83.8 (8.3)	83.9 (8.3)
Sex		
Female	106 080 (71.8)	37 750 (70.7)
Male	41 631 (28.2)	15 623 (29.3)
Race and ethnicity		
Black	5362 (3.6)	1955 (3.7)
Hispanic	1739 (1.2)	572 (1.1)
White	136 461 (92.4)	49 393 (92.6)
Other race^a^	4105 (2.8)	1430 (2.7)
**Clinical characteristics at admission**		
ADL score at admission^b^	18.3 (3.0)	18.3 (3.1)
Moderate to severe cognitive impairment at admission^c^	31 641 (21.5)	12 526 (23.5)
Diabetes	32 945 (22.3)	12 639 (23.7)
Heart failure	21 450 (14.5)	9104 (17.1)
Stroke	6015 (4.1)	3807 (7.1)
Dementia	31 705 (24.8)	12 794 (27.2)
Schizophrenia	1288 (0.9)	497 (0.9)
Bipolar disorder	1681 (1.1)	652 (1.2)
COPD	27 911 (18.9)	11 926 (22.3)
Multiple sclerosis	526 (0.4)	197 (0.4)
Aphasia	1284 (0.9)	762 (1.4)
Dyspnea	16 559 (11.2)	9461 (17.7)
CHESS score, mean (SD)^d^	0.45 (0.69)	0.64 (0.81)

Abbreviations: ADL, activities of daily living; CHESS, Changes in Health, End-stage disease and Symptoms and Signs; COPD, chronic obstructive pulmonary disease.

^a^ Other race includes American Indian or Alaska Native, Asian, and Native Hawaiian or Other Pacific Islander.

^b^ Scores range 0 to 28, with higher scores indicating more functional impairment.

^c^ As determined by a Cognitive Function Scale score of 3 or 4.

^d^ The CHESS score predicts mortality risk and incorporates the following Minimum Data Set indicators: life expectancy, severe cognitive impairment, acute mental status change, aggressive behaviors, impaired decision-making, severe physical impairment, dehydration, pressure ulcers, swallowing disorders, respiratory failure, dyspnea, and heart failure.

### Trends in Clinical Conditions Reported at Admission

Table 1 shows that the average ADL score of admitted SNF patients was roughly the same pre-PDPM and post-PDPM. The proportion of patients admitted with moderate to severe cognitive impairment increased modestly from 21.5% pre-PDPM to 23.5% post-PDPM. The prevalence of most diagnoses increased post-PDPM, with the largest increases noted in diabetes, heart failure, stroke, dementia, chronic obstructive pulmonary disorder, and aphasia. There was also a significant increase in dyspnea prevalence from 11.2% to 17.7% of patients. The mean CHESS score increased from 0.45 to 0.64 (about 30%) post-PDPM. These patterns can also be seen in Figure 1, which shows no trend break in ADL score (B) and proportion with moderate to severe cognitive impairment (C) but a statistically significant increase in mean CHESS score (by 0.1, which is 22% higher than the pre-PDPM mean) post-PDPM implementation (D).

### Trends in Therapy Use

**Figure 2.**
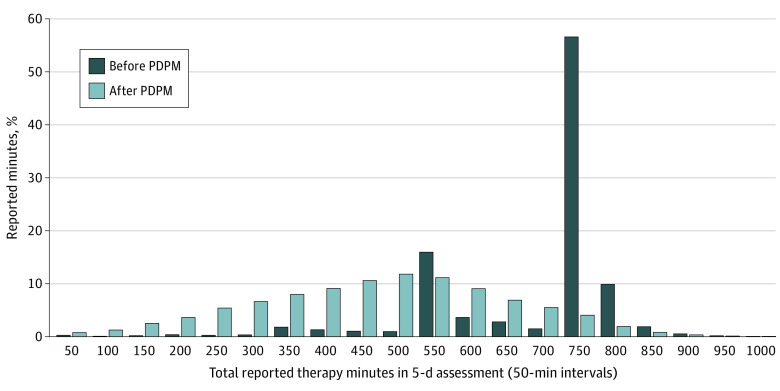
Distribution of Therapy Minutes Provided as Reported in 5-Day Scheduled Assessment for Patients With Hip Fracture Diagnosis Before and After the Patient Driven Payment Model (PDPM) Total therapy use is summation of 3 types of therapy (physical, occupational, and speech) provided at individual or nonindividual sessions. Therapy use is grouped into 50-minute intervals. The x-axis levels show the upper bound of the interval, ie, “50” means 0-50, “100” means 51-100, and so on.

Total therapy use per day in the first week following admission was about 12.3 minutes less for patients admitted under PDPM compared with patients admitted pre-PDPM (Table 2). Figure 2 shows that the distribution of reported therapy minutes changed after the PDPM took effect. Pre-PDPM, we observe 2 sharp spikes, one at 501 to 550 minutes and another at 701 to 750 minutes, consistent with the 500-minute threshold for the high therapy group and the 720-minute threshold for the ultrahigh therapy group under the old RUG system, respectively. More than 55% of patients received just enough therapy to be in the ultrahigh RUG category. By contrast, under the PDPM, the distribution looks bell shaped, with a peak at 451 to 500 minutes.

**Table 2.** Therapy Use and Health Outcomes Following the Adoption of Patient Driven Payment Model (PDPM) for Skilled Nursing Facility Patients With Hip Fracture

Therapy use/outcome	Before PDPM	After PDPM	RD estimate (95% CI)^a^
**Therapy min/d (reported in 5-d scheduled assessment)**			
Individual (physical, occupational, and speech)	96.8	77.8	−15.89 (−16.92 to −14.85)
Nonindividual (physical, occupational, and speech)	0.3	3.0	3.60 (3.38 to 3.83)
Total (physical, occupational, and speech)	97.1	80.8	−12.29 (−13.32 to −11.26)
**Outcomes**			
Any hospital discharge within 40 d of admission, %	19.7	18.3	0.31 (−1.46 to 2.09)
Skilled nursing facility length of stay >40 d, %	42.4	39.9	−2.69 (−4.83 to −0.54)
ADL score at discharge within 40 d, mean (SD)	14.8 (5.3)	14.7 (5.3)	0.04 (−0.19 to 0.26)

Abbreviations: ADL, activities of daily living; RD, regression discontinuity.

^a^ All regressions include age, sex, race and ethnicity, calendar-month (11) dummies, day-of-the-week (6) dummies, and skilled nursing facility fixed effects. Therapy minutes regressions also include count of days between date of admission and 5-day assessment date. The model examining the ADL score at discharge also controls for ADL at admission.

**Figure 3.**
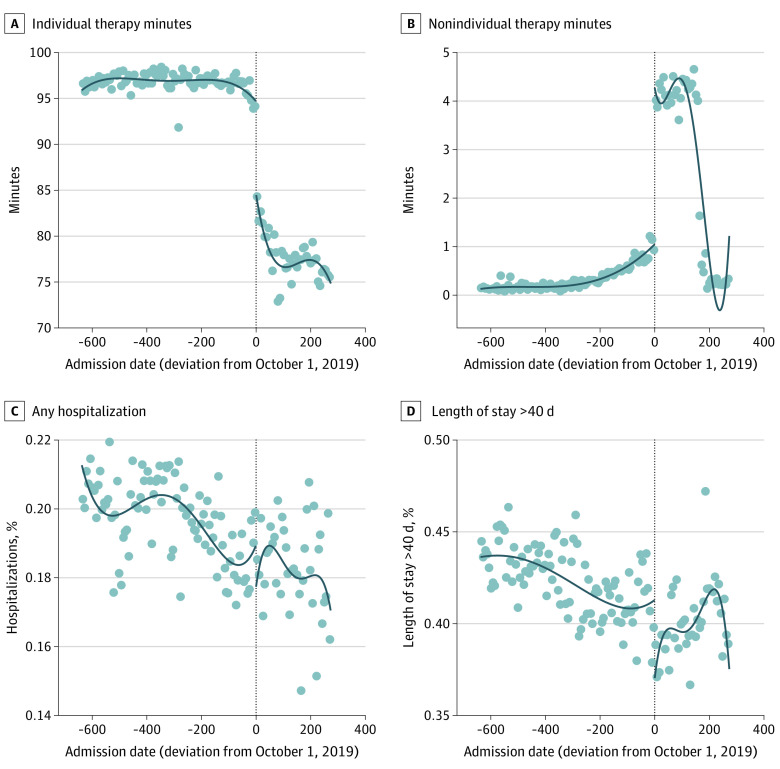
Therapy Use and Health Outcomes Before and After the Patient Driven Payment Model for Patients With a Hip Fracture Diagnosis Each panel is based on a separate regression discontinuity plot. Each scatter dot represents a 7-day average. The lines are based on fourth-degree polynomials.

As shown in Table 2 and Figure 3A, average individual therapy minutes as reported in 5-day assessment declined from 97 minutes per patient per day pre-PDPM to 78 minutes per patient per day post-PDPM implementation (RD estimate: −15.9 minutes per day). On the other hand, nonindividual therapy minutes increased post-PDPM (RD estimate: 3.60 minutes per day). Figure 3B shows the RD time series plot of nonindividual therapy use, which was very low during the earlier months of observation, started increasing pre-PDPM, jumped from about 1 minute per day to 4 minutes per day with PDPM implementation, and then stabilized during the next 5 months before dropping to nearly zero in March 2020, coinciding with the start of the COVID-19 pandemic.

We observed similar trends in therapy use for the overall postacute-care SNF population (eFigure 5 and eTable 2 in the Supplement) as well as in different types of MDS assessments (eFigures 6 and 8 in the Supplement). Additionally, we observed similar trends in different types of SNFs regardless of ownership status, chain affiliation, or the share of Medicare-paid residents (eTable 3 in the Supplement). The RD estimates are robust to different polynomial trend specifications (eTable 4 in the Supplement) and different time window specifications around the PDPM implementation date (eTable 5 in the Supplement).

### Trends in Outcomes

As shown in Table 2, the share of individuals who were discharged directly to the hospital from the SNF within 40 days of admission was 19.7% pre-PDPM and 18.3% post-PDPM (RD estimate: 0.31% chance of discharge post-PDPM; 95% CI, −1.46 to 2.09). Figure 3C shows that the declining trend persisted throughout the study period, and there was no trend break with PDPM implementation.

The share of individuals who remained in SNF for more than 40 days declined from 42.4% pre-PDPM to 39.9% post-PDPM. However, this was mostly owing to the declining trend that was present during the entire study period (Figure 3D). In this plot, we do see a drop in the share of patients with SNF length of stay greater than 40 days following PDPM. Based on the regression results in Table 2, this drop is about 2.7 percentage points (95% CI, −4.83 to −0.54), which is statistically significant at the 5% level of significance but not at 1% level of significance. However, the variance associated with this upward trend is high and probably driven by fewer discharge assessments during the last few weeks of the study period (eFigure 7 in the Supplement). Similarly, we did not observe consistent trends in different types of SNFs in terms of ownership status, chain affiliation, or the share of Medicare-paid residents (eTable 3 in the Supplement). Similarly, the results based on all newly admitted nursing home patients with a 5-day assessment do not point to any robust shift in trend associated with the PDPM in the likelihood of hospitalization or the SNF length of stay lasting more than 40 days (eFigure 5 and eTable 2 in the Supplement). There was no trend break around PDPM implementation in discharge ADL scores (RD estimate: 0.04 ADL score post-PDPM; 95% CI, −0.19 to 0.26) (eFigure 9 in the Supplement).

## Discussion

We found a decrease in total therapy provided as well as a shift in the distribution of therapy minutes following implementation of the PDPM in October 2019. These changes, however, did not appear to be accompanied by consistent changes in key patient outcomes, including rehospitalization, SNF length of stay, or functional scores at time of discharge. We did observe a modest increase in the reporting of certain chronic conditions, despite the overall distribution of age and functional ability among newly admitted SNF patients with hip fracture remaining fairly consistent pre-PDPM and post-PDPM.

The PDPM was designed to be budget neutral and was intended to alter financial incentives for SNFs so that they would provide therapy based on the clinical needs of patients, rather than at levels that maximize Medicare reimbursement. Our findings suggest that the policy has worked as intended. We saw a shift in the distribution of therapy minutes from pre-PDPM, when there were clear spikes at the 500-minute and 720-minute categories, and more than half of patients received just enough therapy minutes to meet the ultrahigh RUG threshold, to post-PDPM where the distribution normalized with a peak at 451 to 500 minutes. This shift in the distribution corresponded with a sharp drop in therapy volume from about 97 minutes to 81 minutes per patient per day with the implementation of the PDPM, strongly suggesting that the shift was driven by the policy change. These conclusions are also supported by recent evidence that SNFs reduced the number of therapy staff they employ or contract with in response to the PDPM.^7^

The reduction in therapy minutes we observed was primarily driven by individual, or 1-on-1, therapy sessions. Nonindividual (ie, group or concurrent) therapy use was uncommon under the RUG system, with a mean of less than 1 minute per patient per day. It did increase under the PDPM to an average of 3 minutes per day, but this was still less than the individual therapy average of about 78 minutes per day, with most patients receiving zero minutes of nonindividual therapy. We also observed a drop-off in nonindividual therapy minutes in March 2020 with the onset of the COVID-19 pandemic, which resulted in SNFs severely limiting or eliminating communal activities in order to reduce virus transmission. It will be important to follow these trends over time to determine whether SNFs increase their use of nonindividual therapy in the aftermath of the pandemic.

We limited our primary analysis to patients admitted to SNFs with hip fracture to isolate the association between PDPM and therapy use and reduce potential confounding caused by SNFs admitting a different case mix of patients pre-PDPM vs post-PDPM. Because per diem reimbursement rates are now determined based on the clinical acuity of patients at time of admission, SNFs may be incentivized to admit more medically complex patients to boost reimbursement. Patients with hip fracture are less heterogeneous than the SNF population overall and generally have high therapy needs on hospital discharge. We observed that the age and functional characteristics of newly admitted patients with hip fracture remained relatively unchanged pre-PDPM and post-PDPM; however, we did observe an increase in certain chronic conditions following PDPM implementation. The discontinuous jump in CHESS scores is consistent with an increase in coding intensity of chronic conditions to improve reimbursement. Future work to explore these questions will need to examine how well case mix measured from SNF records aligns with case mix measured from the preceding hospital stay once Medicare claims for the study period become available.

The ultimate question is whether the reduction in the therapy volume is associated with worse patient outcomes. This did not appear to be the case with regard to 3 key outcomes (rehospitalization, SNF length of stay, and functional scores at time of discharge), as no significant changes were found. This suggests that the PDPM resulted in a “right-sizing” of therapy provision in SNFs and reversed prior incentives under the RUG system to provide therapy when there was minimal clinical benefit. One caveat of our rehospitalization finding, however, is that it is not risk adjusted. We did not include acuity measures while comparing outcomes before and after PDPM because changes in coding and reported acuity (shown by higher CHESS scores) following PDPM are likely attributable to bias risk adjustment and subsequently make post-PDPM risk-adjusted hospitalization rates look lower than they would without adjustment for clinical complexity. Prior studies have shown that when payment policy changes drive changes in coding intensity, it can complicate trend analyses of risk-adjusted outcomes.^15,16^

### Limitations

A few limitations of the study should be noted. First, lack of claims data reduces precision of our cohort selection and outcome definitions. Our hospitalization outcome measure is not a typical hospital readmission measure, which requires an index hospital claim. We relied on the presence of 5-day assessment, which is typically performed for postacute-care patients. Second, lack of claims data also limits our ability to understand whether an increase in MDS-reported chronic conditions reflects a true increase in patient complexity or an increase in coding intensity. Finally, the fact that CMS reduced the required frequency of MDS assessments under the PDPM compared with the RUG system prevents us from comparing therapy use and ADL score changes over the duration of the SNF stay and limits us to using the admission and discharge assessments.

## Conclusions

In this cross-sectional study of patients admitted to an SNF after hip fracture between January 2018 and March 2020, we observed a significant reduction in therapy volume and a shift in the distribution of therapy minutes away from levels that previously resulted in maximum reimbursement under the RUG system. Despite these changes, we observed no robust differences in 3 key patient outcomes: rehospitalization, SNF length of stay, and functional scores at discharge. These findings suggest that the PDPM did have its intended effect of altering financial incentives for SNFs related to therapy provision; however, future study is needed to refine the measurement of patient outcomes and examine potential changes in case mix.

## Notes

Supplement. **eAppendix.** STROBE Statement—Checklist of items that should be included in reports of cohort studies **eTable 1.** Sample selection flow **eTable 2.** Therapy use and health outcomes following the adoption of Patient Driven Payment Model (PDPM) for all skilled nursing facility patients with a 5-day assessment **eTable 3.** Estimated effect of PDPM for different types of skilled nursing facilities **eTable 4.** Regression discontinuity estimate using different polynomials **eTable 5.** Regression discontinuity estimates shorter time windows around PDPM **eFigure 1.** Number of hip fracture admissions per day **eFigure 2.** Proportion of newly admitted nursing elderly nursing home patients enrolled in FFS with hip fracture diagnosis who had a 5-day scheduled assessment and were included in our primary analysis **eFigure 3.** Days between admission and 5-day schedule assessment **eFigure 4.** Number of Minimum Data Set (MDS) assessments in 40 days following admission **eFigure 5.** Therapy use and health outcomes before and under the Patient Driven Payment Model (PDPM) for all newly admitted skilled nursing facility patients with 5-day assessment **eFigure 6.** Trajectory of Therapy use before and under the Patient Driven Payment Model (PDPM) **eFigure 7.** Proportion of individuals with a discharge assessment within 40 days of admission **eFigure 8.** Reported therapy use in 5-day and discharge assessment before and under Patient Driven Payment Model (PDPM) **eFigure 9.** Changes in Activities of Daily Living (ADL) scores at admission and discharge before and under Patient Driven Payment Model (PDPM)
