# Supplementary material for: Association Between the Patient Driven Payment Model and Therapy Utilization and Patient Outcomes in US Skilled Nursing Facilities
Source: JAMA Health Forum. 2022 Jan 7;3(1):e214366. doi: 10.1001/jamahealthforum.2021.4366 (PMC8903117; doi:10.1001/jamahealthforum.2021.4366)
Supplement: Supplement. — eAppendix. STROBE Statement—Checklist of items that should be included in reports of cohort studies eTable 1. Sample selection flow eTable 2. Therapy use and health outcomes following the adoption of Patient Driven Payment Model (PDPM) for all skilled nursing facility patients with a 5-day assessment eTable 3. Estimated effect of PDPM for different types of skilled nursing facilities eTable 4. Regression discontinuity estimate using different polynomials eTable 5. Regression discontinuity estimates shorter time windows around PDPM eFigure 1. Number of hip fracture admissions per day eFigure 2. Proportion of newly admitted nursing elderly nursing home patients enrolled in FFS with hip fracture diagnosis who had a 5-day scheduled assessment and were included in our primary analysis eFigure 3. Days between admission and 5-day schedule assessment eFigure 4. Number of Minimum Data Set (MDS) assessments in 40 days following admission eFigure 5. Therapy use and health outcomes before and under the Patient Driven Payment Model (PDPM) for all newly admitted skilled nursing facility patients with 5-day assessment eFigure 6. Trajectory of Therapy use before and under the Patient Driven Payment Model (PDPM) eFigure 7. Proportion of individuals with a discharge assessment within 40 days of admission eFigure 8. Reported therapy use in 5-day and discharge assessment before and under Patient Driven Payment Model (PDPM) eFigure 9. Changes in Activities of Daily Living (ADL) scores at admission and discharge before and under Patient Driven Payment Model (PDPM) [file jamahealthforum-e214366-s001.pdf]

Supplemental Online Content

Rahman M, White EM, McGarry BE, et al. Association between the Patient Driven Payment Model and therapy utilization and patient outcomes in US skilled nursing facilities. *JAMA Health Forum*. 2022;3(1):e214366. doi:10.1001/jamahealthforum.2021.4366

eAppendix. STROBE Statement—Checklist of items that should be included in reports of cohort studies

eTable 1. Sample selection flow

eTable 2. Therapy use and health outcomes following the adoption of Patient Driven Payment Model (PDPM) for all skilled nursing facility patients with a 5-day assessment

eTable 3. Estimated effect of PDPM for different types of skilled nursing facilities

eTable 4. Regression discontinuity estimate using different polynomials

eTable 5. Regression discontinuity estimates shorter time windows around PDPM

eFigure 1. Number of hip fracture admissions per day

eFigure 2. Proportion of newly admitted nursing elderly nursing home patients enrolled in FFS with hip fracture diagnosis who had a 5-day scheduled assessment and were included in our primary analysis

eFigure 3. Days between admission and 5-day schedule assessment

eFigure 4. Number of Minimum Data Set (MDS) assessments in 40 days following admission

eFigure 5. Therapy use and health outcomes before and under the Patient Driven Payment Model (PDPM) for all newly admitted skilled nursing facility patients with 5-day assessment

eFigure 6. Trajectory of Therapy use before and under the Patient Driven Payment Model (PDPM)

eFigure 7. Proportion of individuals with a discharge assessment within 40 days of admission

eFigure 8. Reported therapy use in 5-day and discharge assessment before and under Patient Driven Payment Model (PDPM)

eFigure 9. Changes in Activities of Daily Living (ADL) scores at admission and discharge before and under Patient Driven Payment Model (PDPM)

This supplemental material has been provided by the authors to give readers additional information about their work.

eAppendix. STROBE Statement—Checklist of items that should be included in reports of *cohort studies*

	Item No	Recommendation	Page No
**Title and abstract**	1	(*a*) Indicate the study's design with a commonly used term in the title or the abstract	2
		(*b*) Provide in the abstract an informative and balanced summary of what was done and what was found	2
**Introduction**			
Background/rationale	2	Explain the scientific background and rationale for the investigation being reported	4
Objectives	3	State specific objectives, including any prespecified hypotheses	5
**Methods**			
Study design	4	Present key elements of study design early in the paper	5
Setting	5	Describe the setting, locations, and relevant dates, including periods of recruitment, exposure, follow-up, and data collection	6
Participants	6	(*a*) Give the eligibility criteria, and the sources and methods of selection of participants. Describe methods of follow-up	6
		(*b*) For matched studies, give matching criteria and number of exposed and unexposed	NA
Variables	7	Clearly define all outcomes, exposures, predictors, potential confounders, and effect modifiers. Give diagnostic criteria, if applicable	6-7
Data sources/ measurement	8*	For each variable of interest, give sources of data and details of methods of assessment (measurement). Describe comparability of assessment methods if there is more than one group	5
Bias	9	Describe any efforts to address potential sources of bias	9
Study size	10	Explain how the study size was arrived at	8
Quantitative variables	11	Explain how quantitative variables were handled in the analyses. If applicable, describe which groupings were chosen and why	7
Statistical methods	12	(*a*) Describe all statistical methods, including those used to control for confounding	7-9
		(*b*) Describe any methods used to examine subgroups and interactions	9
		(*c*) Explain how missing data were addressed	8
		(*d*) If applicable, explain how loss to follow-up was addressed	NA
		(*e*) Describe any sensitivity analyses	9
**Results**			
Participants	13*	(a) Report numbers of individuals at each stage of study—eg numbers potentially eligible, examined for eligibility, confirmed eligible, included in the study, completing follow-up, and analysed	9-10
		(b) Give reasons for non-participation at each stage	NA
		(c) Consider use of a flow diagram	NA
Descriptive data	14*	(a) Give characteristics of study participants (eg demographic, clinical, social) and information on exposures and potential confounders	10-12
		(b) Indicate number of participants with missing data for each variable of interest	Table 1
		(c) Summarise follow-up time (eg, average and total amount)	11-12
Outcome data	15*	Report numbers of outcome events or summary measures over time	12-13

Main results	16	(*a*) Give unadjusted estimates and, if applicable, confounder-adjusted estimates and their precision (eg, 95% confidence interval). Make clear which confounders were adjusted for and why they were included	10-13
		(*b*) Report category boundaries when continuous variables were categorized	10-13
		(*c*) If relevant, consider translating estimates of relative risk into absolute risk for a meaningful time period	NA
Other analyses	17	Report other analyses done—eg analyses of subgroups and interactions, and sensitivity analyses	13
**Discussion**			
Key results	18	Summarise key results with reference to study objectives	14
Limitations	19	Discuss limitations of the study, taking into account sources of potential bias or imprecision. Discuss both direction and magnitude of any potential bias	15-16
Interpretation	20	Give a cautious overall interpretation of results considering objectives, limitations, multiplicity of analyses, results from similar studies, and other relevant evidence	14-16
Generalisability	21	Discuss the generalisability (external validity) of the study results	16
**Other information**			
Funding	22	Give the source of funding and the role of the funders for the present study and, if applicable, for the original study on which the present article is based	Title page

*Give information separately for exposed and unexposed groups.

Note: An Explanation and Elaboration article discusses each checklist item and gives methodological background and published examples of transparent reporting. The STROBE checklist is best used in conjunction with this article (freely available on the Web sites of PLoS Medicine at http://www.plosmedicine.org/, Annals of Internal Medicine at http://www.annals.org/, and Epidemiology at http://www.epidem.com/). Information on the STROBE Initiative is available at http://www.strobe-statement.org.

© 2022 Rahman M et al. *JAMA Health Forum.*

eTable 1. Sample selection flow

Inclusion criteria	Remaining sample	
	Before-PDPM	PDPM
Newly admitted SNF patients between 1/1/2018 – 6/30/2020	4,055,810	1,414,851
Age >=65	3,396,012	1,185,452
Have 5-day scheduled assessment	1,714,969	590,995
Fee-for-service Medicare enrollment	1,565,207	534,927
Have hip-fracture diagnosis	147,711	53,373

© 2022 Rahman M et al. *JAMA Health Forum.*

eTable 2. Therapy use and health outcomes following the adoption of Patient Driven Payment Model (PDPM) for all skilled nursing facility patients with a 5-day assessment

		Before-PDPM	PDPM	Regression discontinuity Estimate
Therapy minutes per day (reported in 5-day scheduled assessment)	Individual (physical, occupational and speech)	94.4	74.7	-14.5***<br>[-14.9 - -14.1]
	Non-individual (physical, occupational and speech)	0.4	4.0	3.1***<br>[3.0 – 3.3]
	Total (physical, occupational and speech)	94.8	78.6	-11.4***<br>[-11.8 - -11.0]
Outcomes	Any hospital discharge within 40 days of admission	23.8%	21.3%	0.52*<br>[-0.02 – 1.1]
	Skilled nursing facility length of stay greater than 40 days	34.3%	33.5%	-0.26<br>[-0.84 – 0.31]
	Activities of Daily Living score at discharge within 40 days	13.8	13.7	0.11***<br>[0.05 – 0.17]

Notes: All regressions include age, gender, race, calendar month (11) dummies, day of the week (6) dummies and skilled nursing facility fixed effects. Therapy minutes regressions also include count of days between date of admission and 5-day assessment date. The model examining the Activities of Daily Living (ADL) score at discharge also controls for ADL at admission.

© 2022 Rahman M et al. *JAMA Health Forum.*

eTable 3. Estimated effect of PDPM for different types of skilled nursing facilities

SNF characteristics		Individual (physical, occupational and speech)	Non-individual (physical, occupational and speech)	Total (physical, occupational and speech)	Any hospital discharge within 40 days of admission	Skilled nursing facility length of stay greater than 40 days	Activities of Daily Living score at discharge within 40 days
Profit status	Not for profit	-14.39<br>(-16.17 - -12.61)	3.533<br>(3.135 - 3.930)	-10.86<br>(-12.62 - -9.104)	1.123<br>(-1.766 - 4.011)	-2.929<br>(-6.495 - 0.636)	0.109<br>(-0.271 - 0.490)
	For profit	-16.71<br>(-18.03 - -15.40)	3.662<br>(3.379 - 3.946)	-13.05<br>(-14.37 - -11.74)	-0.0255<br>(-2.348 - 2.297)	-2.649<br>(-5.425 - 0.126)	-0.0549<br>(-0.346 - 0.236)
Chain status	Free standing	-16.31<br>(-17.94 - -14.67)	3.430<br>(3.074 - 3.787)	-12.88<br>(-14.52 - -11.24)	0.595<br>(-2.201 - 3.391)	-2.771<br>(-6.124 - 0.582)	-0.0462<br>(-0.406 - 0.314)
	Part of chain	-15.37<br>(-16.76 - -13.98)	3.745<br>(3.441 - 4.050)	-11.63<br>(-13.00 - -10.26)	0.237<br>(-2.146 - 2.620)	-2.788<br>(-5.686 - 0.110)	0.0392<br>(-0.262 - 0.341)
Share of Medicare paid patients	<30%	-15.37<br>(-16.59 - -14.15)	3.550<br>(3.288 - 3.813)	-11.83<br>(-13.04 - -10.61)	-0.082<br>(-2.232 - 2.068)	-2.387<br>(-4.978 - 0.204)	0.0338<br>(-0.248 - 0.315)
	>=30%	-16.89<br>(-19.03 - -14.76)	3.791<br>(3.310 - 4.272)	-13.11<br>(-15.23 - -10.99)	1.636<br>(-1.716 - 4.987)	-3.961<br>(-8.058 - 0.135)	-0.061<br>(-0.464 - 0.342)

Notes: All regressions include age, gender, race, calendar month (11) dummies, day of the week (6) dummies and skilled nursing facility fixed effects. Therapy minutes regressions also include count of days between date of admission and 5-day assessment date. The model examining the Activities of Daily Living (ADL) score at discharge also controls for ADL at admission. Robust confidence intervals in parentheses.

© 2022 Rahman M et al. *JAMA Health Forum.*

eTable 4. Regression discontinuity estimate using different polynomials

		Linear	Third degree polynomial	5th degree polynomial
Therapy minutes per day (reported in 5-day scheduled assessment)	Individual (physical, occupational and speech)	-21.48 (-22.08 - -20.88)	-18.50 (-19.32 - -17.69)	-15.88 (-16.92 - -14.84)
	Non-individual (physical, occupational and speech)	2.631 (2.504 - 2.757)	5.10 (4.895 - 5.304)	3.635 (3.408 - 3.863)
	Total (physical, occupational and speech)	-18.85 (-19.44 - -18.27)	-13.41 (-14.21 - -12.61)	-12.25 (-13.28 - -11.21)
Outcomes	Any hospital discharge within 40 days of admission	-0.528 (-1.272 - 0.217)	-0.24 (-1.445 - 0.966)	0.314 (-1.462 - 2.090)
	Skilled nursing facility length of stay greater than 40 days	0.433 (-0.485 - 1.351)	-1.454 (-2.906 - -0.001)	-2.755 (-4.902 - -0.609)
	Activities of Daily Living score at discharge within 40 days	0.0364 (-0.0624 - 0.135)	-0.0136 (-0.168 - 0.141)	0.0314 (-0.195 - 0.258)

Notes: All regressions include age, gender, race, calendar month (11) dummies, day of the week (6) dummies and skilled nursing facility fixed effects. Therapy minutes regressions also include count of days between date of admission and 5-day assessment date. The model examining the Activities of Daily Living (ADL) score at discharge also controls for ADL at admission. Robust confidence intervals in parentheses.

© 2022 Rahman M et al. *JAMA Health Forum.*

eTable 5. Regression discontinuity estimates shorter time windows around PDPM

		Based on nine months before and after PDPM	Based on six months before and after PDPM
N		114,559	78,421
Therapy minutes per day (reported in 5-day scheduled assessment) Outcomes	Individual (physical, occupational and speech) Non-individual (physical, occupational and speech)	-16.11	-13.03
		(-17.01 - -15.21)	(-14.19 - -11.88)
	Total (physical, occupational and speech) Any hospital discharge within 40 days of admission	3.662	2.618
		(3.435 - 3.890)	(2.362 - 2.875)
	Skilled nursing facility length of stay greater than 40 days	-12.45	-10.42
		(-13.34 - -11.56)	(-11.58 - -9.262)
Therapy minutes per day (reported in 5-day scheduled assessment)	Individual (physical, occupational and speech) Non-individual (physical, occupational and speech)	0.302	0.0972
		(-1.004 - 1.609)	(-1.571 - 1.765)
	Total (physical, occupational and speech) Any hospital discharge within 40 days of admission	-2.573	-2.324
		(-4.196 - -0.949)	(-4.343 - -0.305)
	Skilled nursing facility length of stay greater than 40 days	0.119	0.213
		(-0.0491 - 0.288)	(-0.00464 - 0.431)

Notes: All regressions include age, gender, race, day of the week (6) dummies and skilled nursing facility fixed effects. Therapy minutes regressions also include count of days between date of admission and 5-day assessment date. The model examining the Activities of Daily Living (ADL) score at discharge also controls for ADL at admission. Robust confidence intervals in parentheses.

© 2022 Rahman M et al. *JAMA Health Forum.*

eFigure 1. Number of hip fracture admissions per day

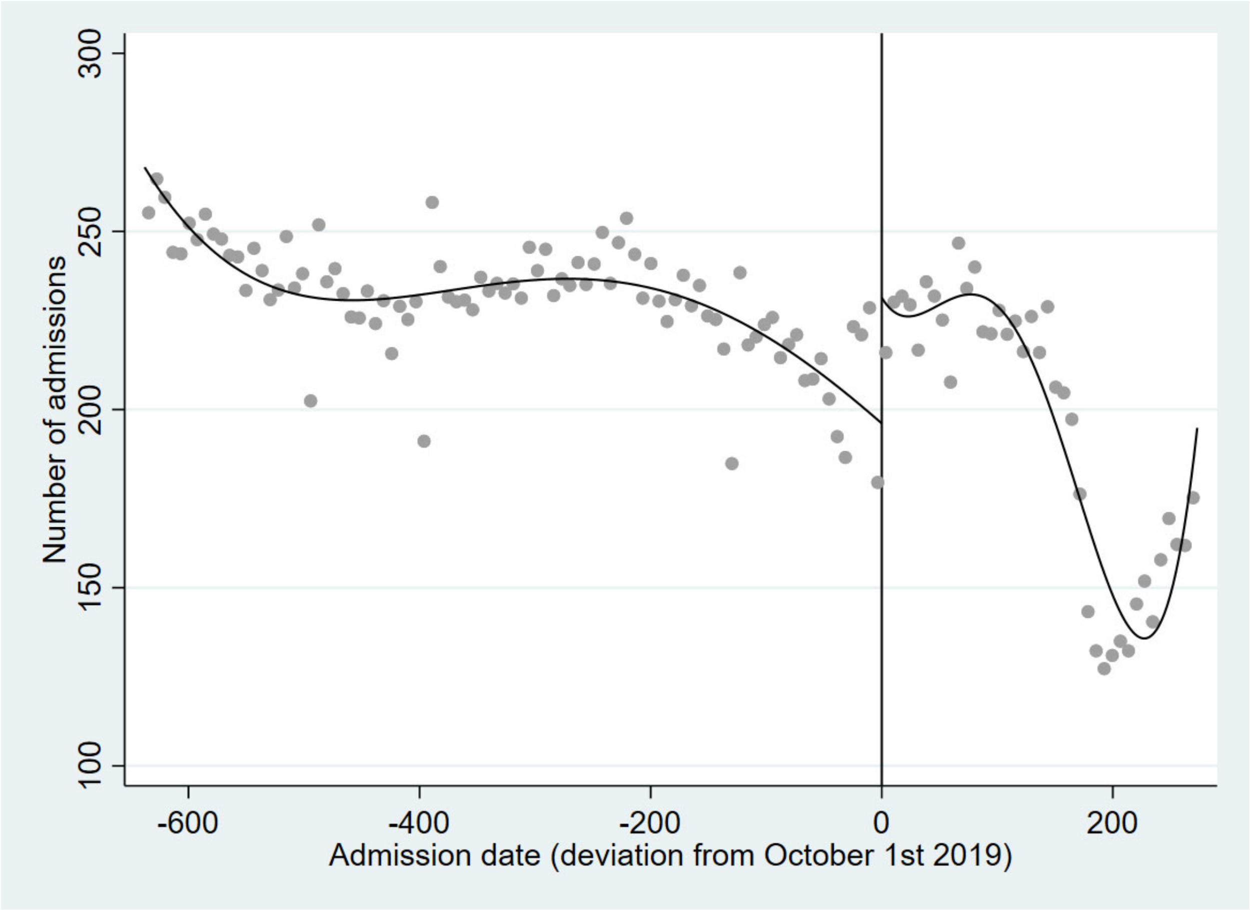

Notes: This figure is based on 201,184 elderly Medicare beneficiaries who were newly admitted to a skilled nursing facility with a hip fracture and had a 5-day scheduled assessment. We collapsed the individual level data to daily level to calculate daily number of admissions and then created the regression discontinuity plot.

© 2022 Rahman M et al. *JAMA Health Forum.*

eFigure 2. Proportion of newly admitted nursing elderly nursing home patients enrolled in FFS with hip fracture diagnosis who had a 5-day scheduled assessment and were included in our primary analysis

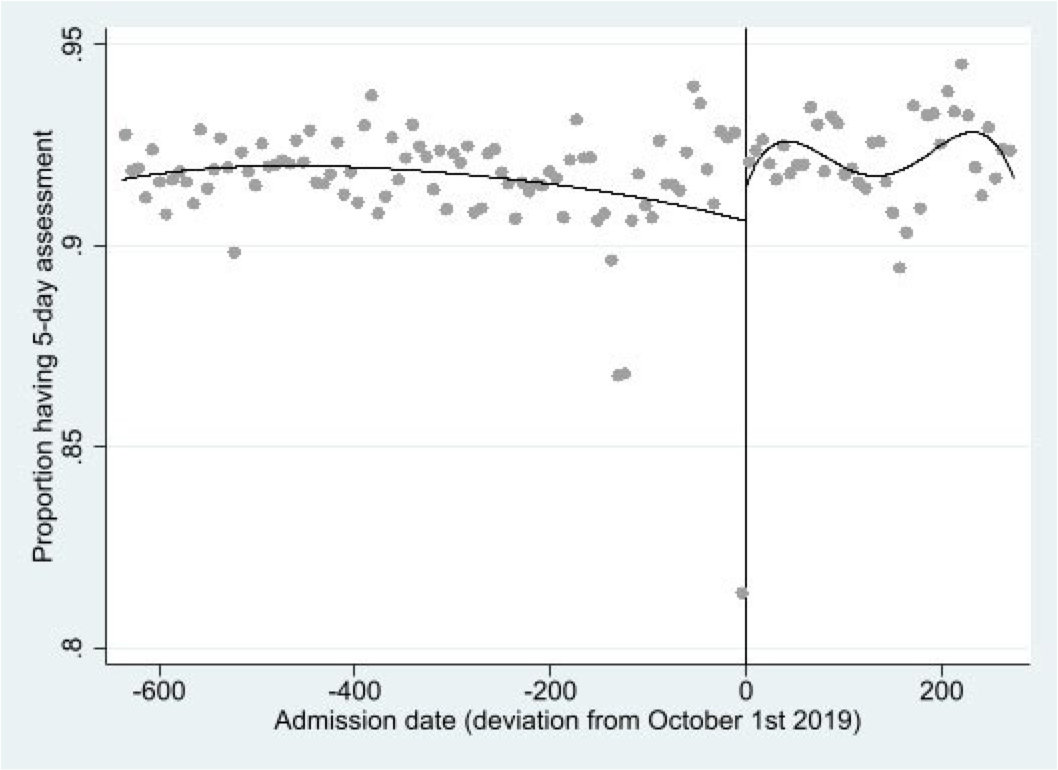

Notes: This figure is based on 201,184 (147,711 before the Patient Driven Payment Model and 53,373 under the Patient Driven Payment Model) elderly Medicare beneficiaries who were newly admitted to a skilled nursing facility with a hip fracture.

© 2022 Rahman M et al. *JAMA Health Forum.*

eFigure 3. Days between admission and 5-day schedule assessment

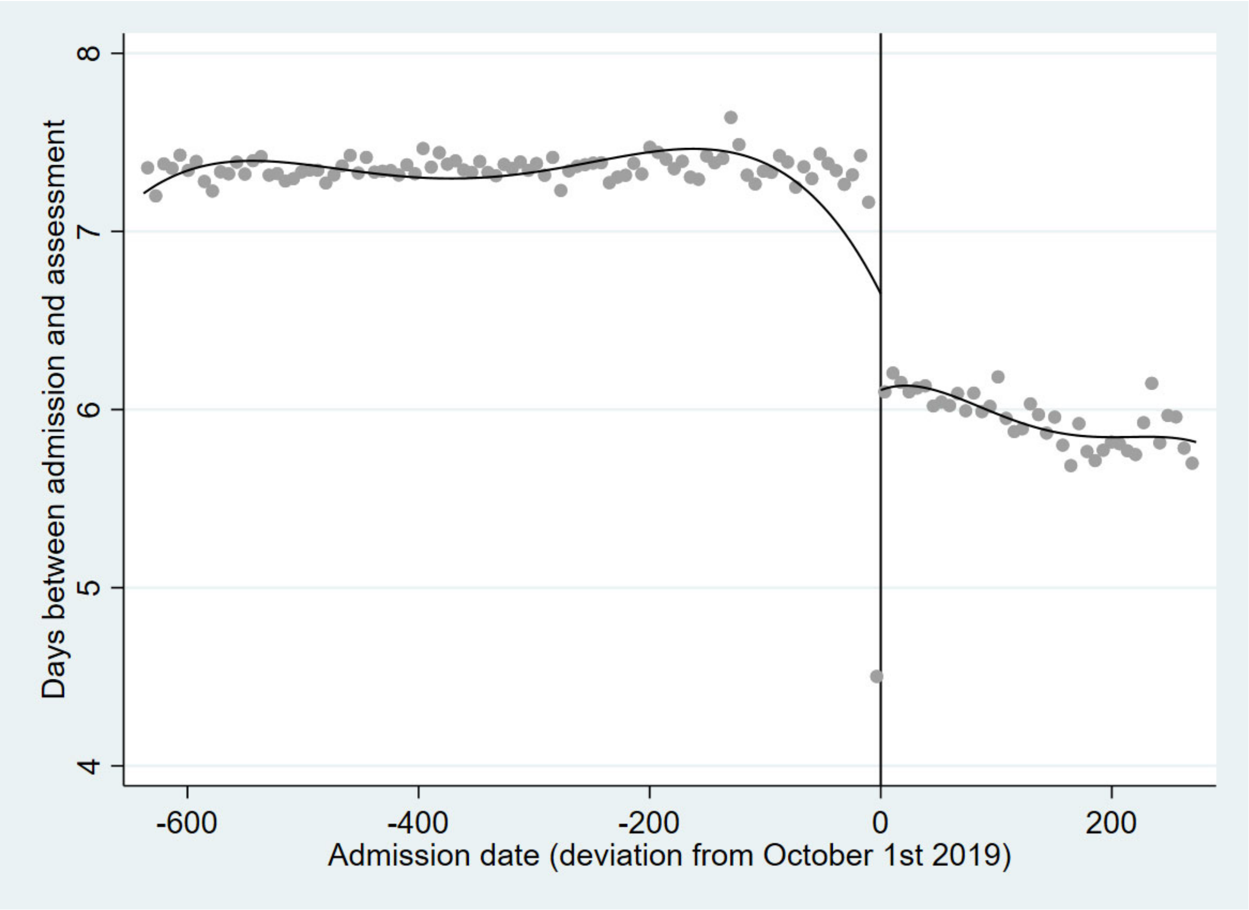

Notes: This figure is based on 201,184 elderly Medicare beneficiaries who were newly admitted to a skilled nursing facility with a hip fracture and had a 5-day scheduled assessment.

© 2022 Rahman M et al. *JAMA Health Forum.*

eFigure 4. Number of Minimum Data Set (MDS) assessments in 40 days following admission

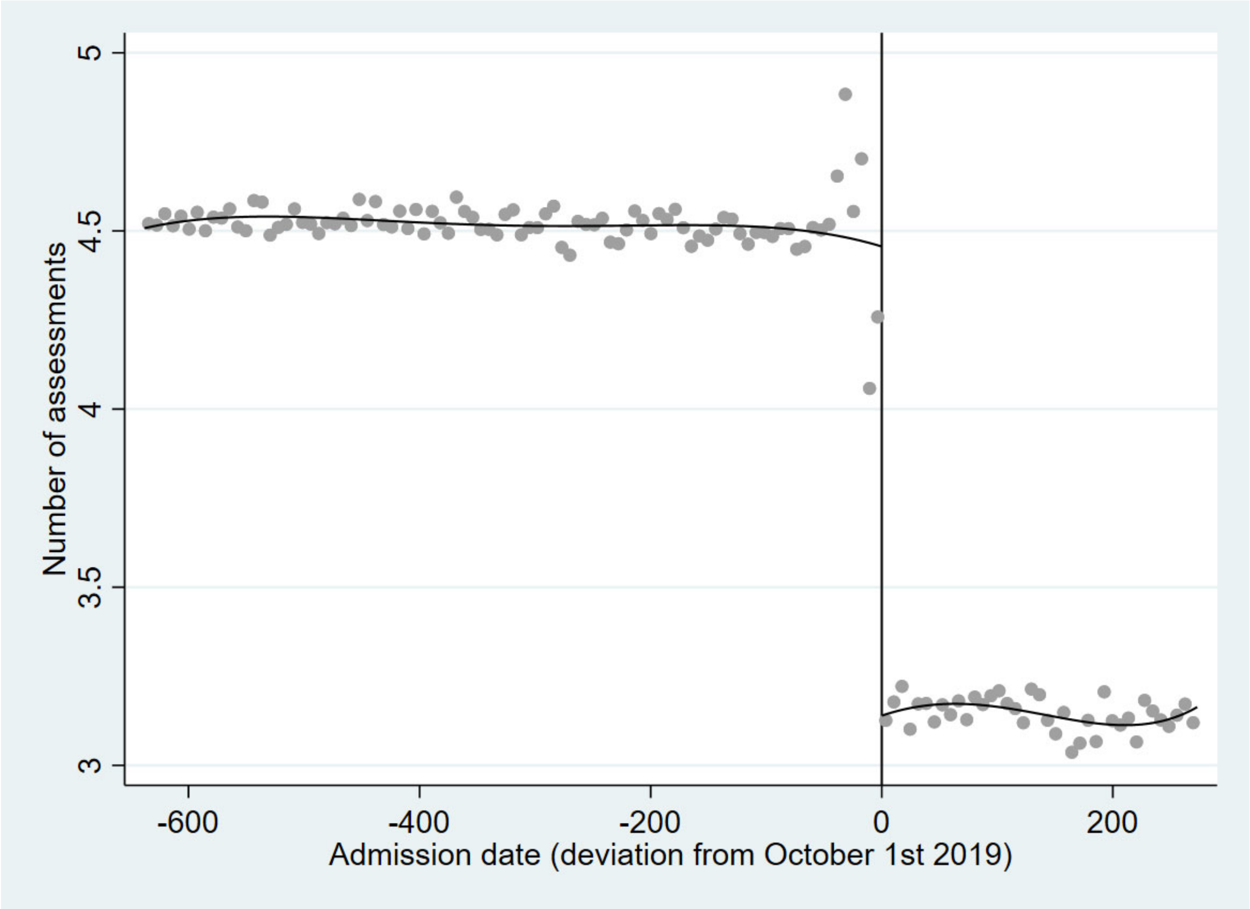

Notes: The average number of assessments in the 40 days following admission dropped from 4.7 before the Patient Driven Payment Model (PDPM) to 3.3 under PDPM. The PDPM mandates fewer MDS assessments than was required under the Resource Utilization Group system.

© 2022 Rahman M et al. *JAMA Health Forum.*

eFigure 5. Therapy use and health outcomes before and under the Patient Driven Payment Model (PDPM) for all newly admitted skilled nursing facility patients with 5-day assessment

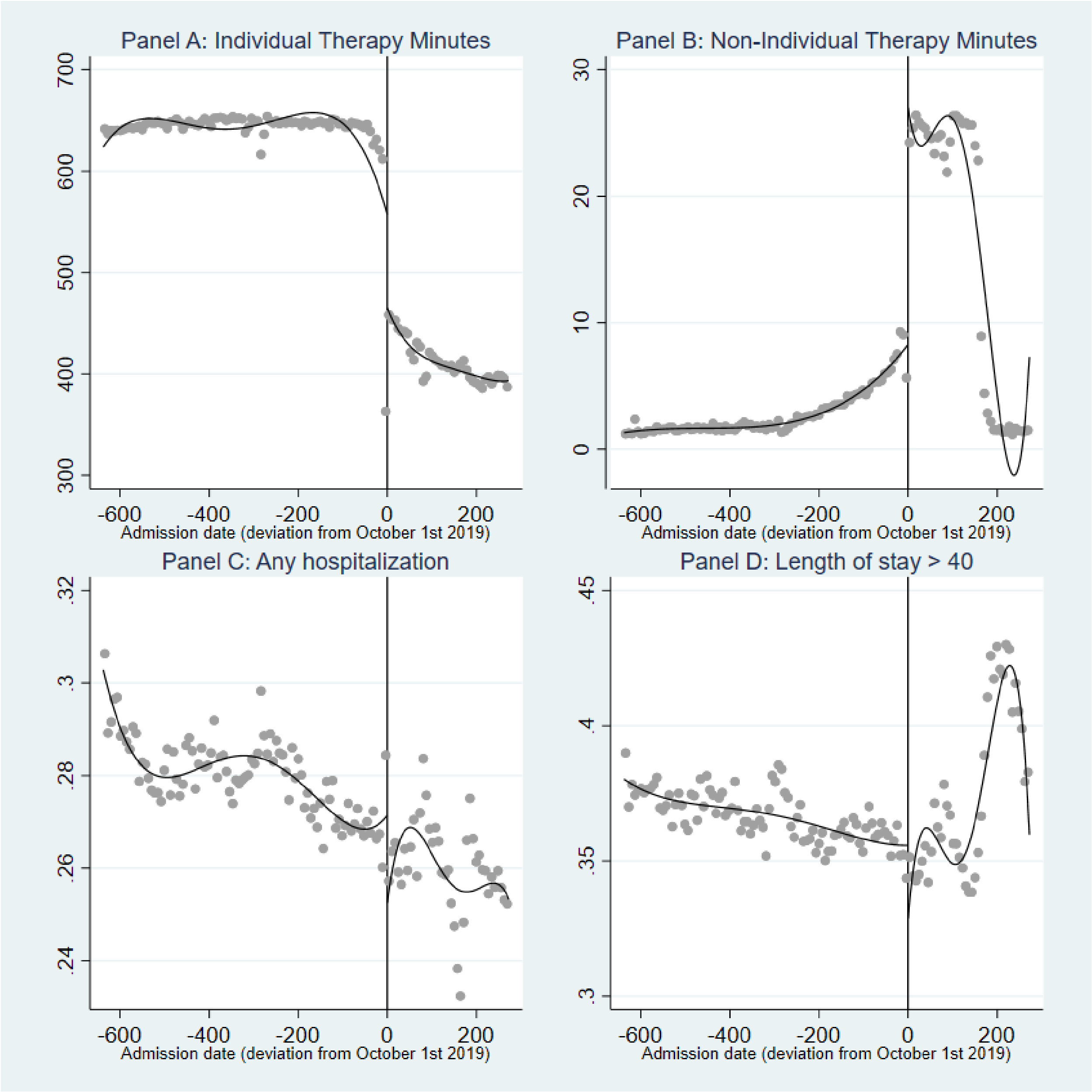

© 2022 Rahman M et al. *JAMA Health Forum.*

eFigure 6. Trajectory of Therapy use before and under the Patient Driven Payment Model (PDPM)

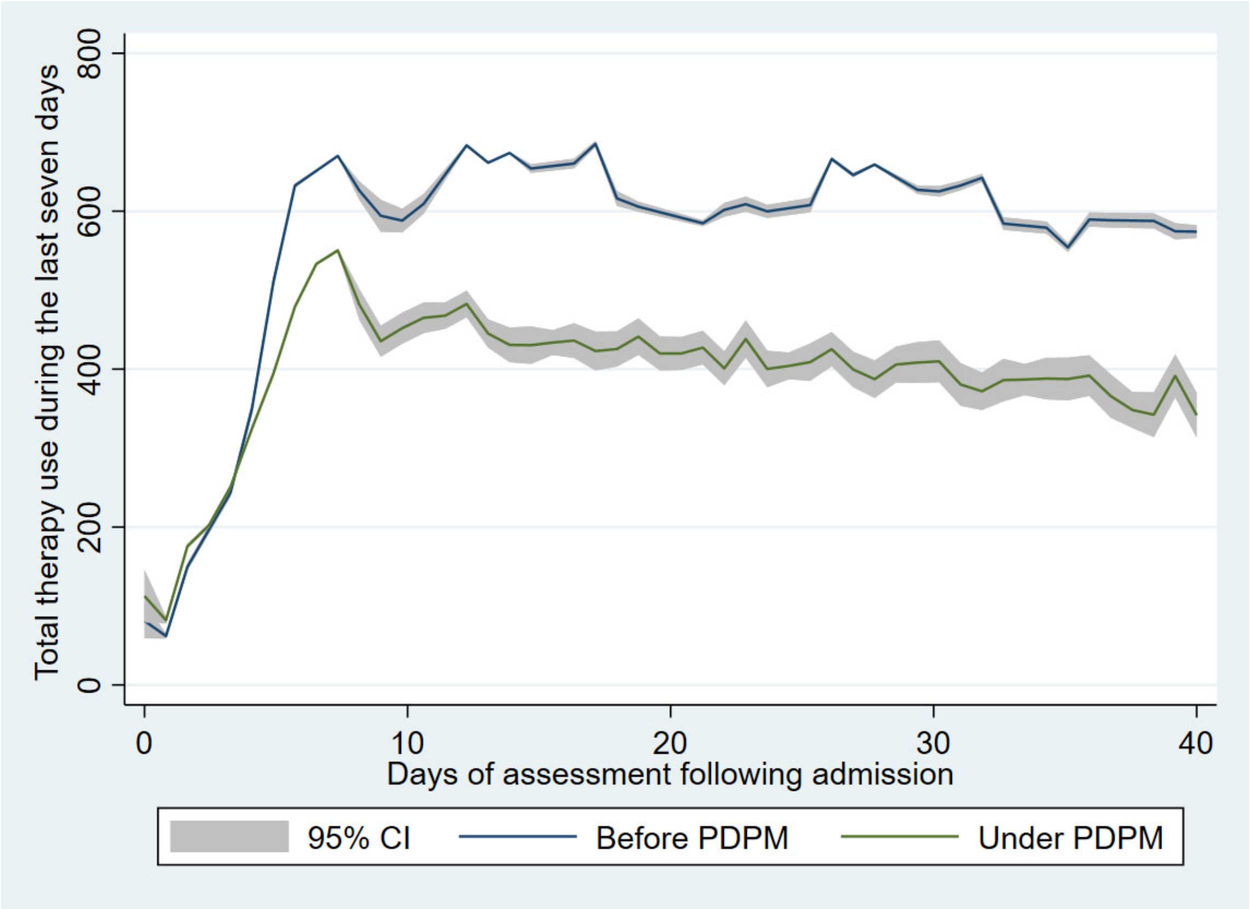

© 2022 Rahman M et al. *JAMA Health Forum.*

eFigure 7. Proportion of individuals with a discharge assessment within 40 days of admission

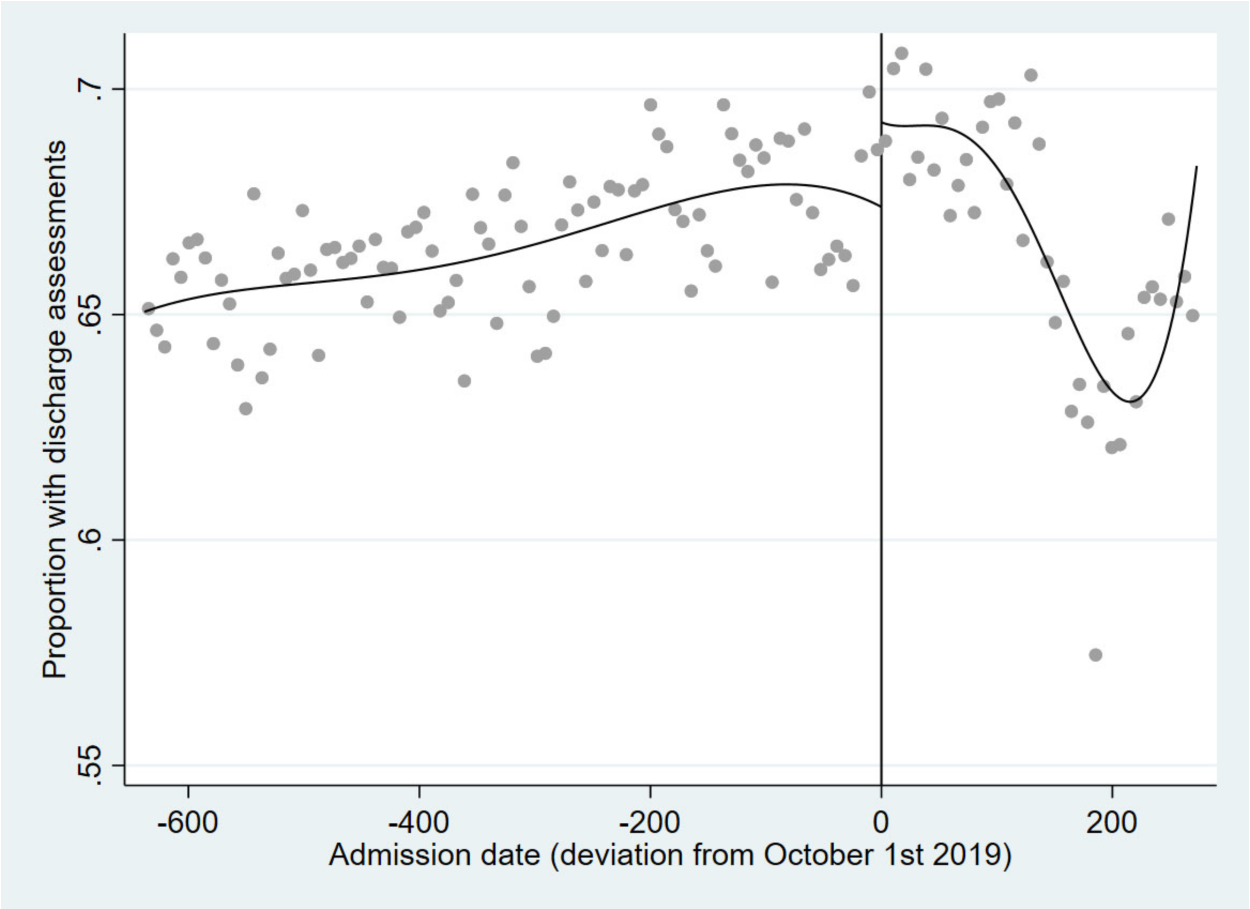

© 2022 Rahman M et al. *JAMA Health Forum.*

eFigure 8. Reported therapy use in 5-day and discharge assessment before and under Patient Driven Payment Model (PDPM)

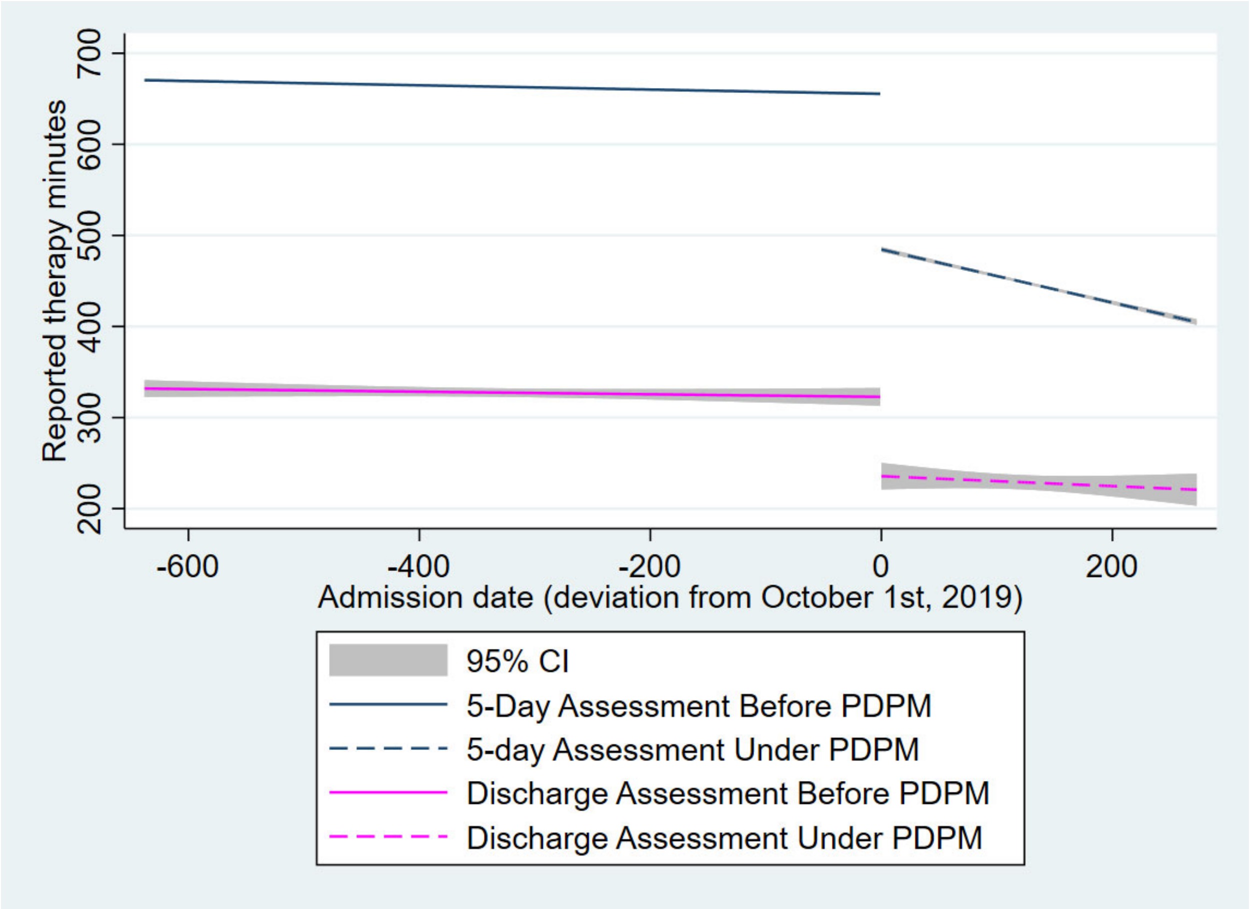

© 2022 Rahman M et al. *JAMA Health Forum.*

eFigure 9. Changes in Activities of Daily Living (ADL) scores at admission and discharge before and under Patient Driven Payment Model (PDPM)

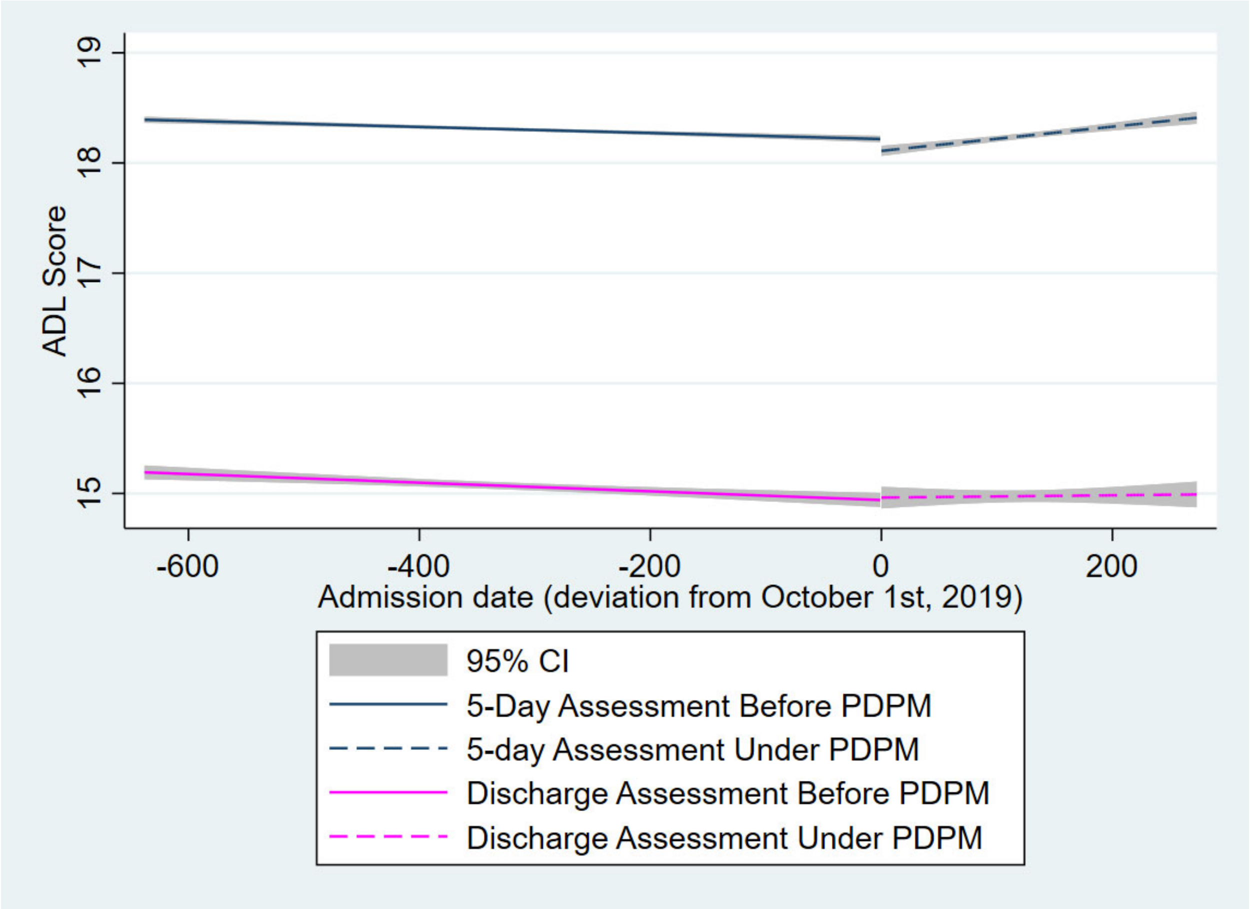

Notes: The Morris Activities of Daily Living score ranges from 0-28 with higher scores indicating greater functional impairment

© 2022 Rahman M et al. *JAMA Health Forum.*
